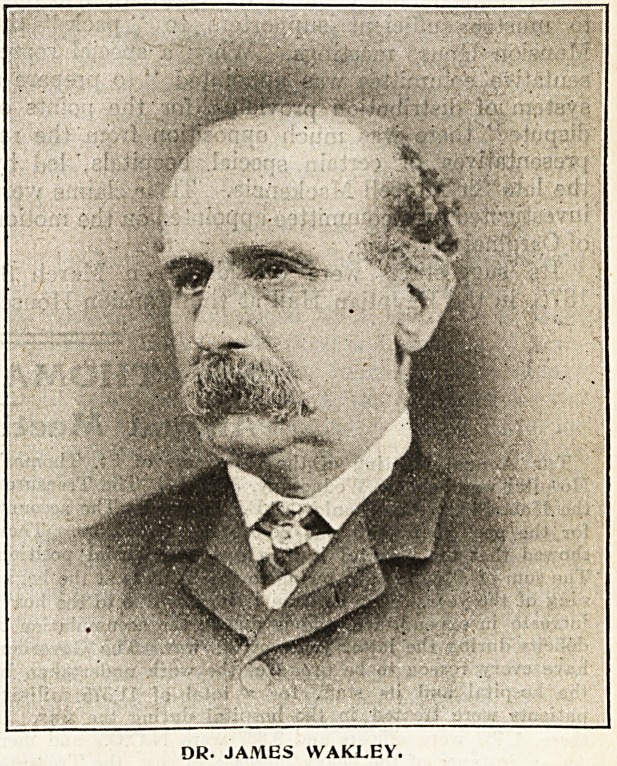# Great Men and Movements of the past

**Published:** 1920-06-26

**Authors:** 


### THE STORY OF HOSPITAL SUNDAY.

### Great Men and Movements o the Past.

Though the idea of simultaneous collections for hospitals and dispensaries is said to have been carried out in Manchester as early as the year 1792, the foundation of the Hospital Sunday Fund movement must be credited to the late Canon J. C. Miller, Vicar and Rural Dean of Greenwich and Canon of Rochester. At a meeting of ministers of all denominations which was held in Birmingham Town Hall on October 25, 1859, Dr. Miller, who presided, acknowledged that it was an article in the *Midland Counties Herald*, published on October 13, 1859, which had inspired him. The paper has recorded that the article was "prepared for the *Herald* by the direction of Mr. Thomas Barber Wright (its editor), by the late Mr. Davidson." Mr. Wright's suggestion was that "congregational collections should be made in all places of worship in the town and district annually." This inspired Dr. Miller to take the lead and to realise the intention.

CANON MILLER.
Founder of Hospital Sunday.

### Canon Miller's Story.

When Dr. Miller died, at the age of sixty-five, in 1880, a memoir was published by the *Kentish Mercury*, later reprinted in a pamphlet. It described him to be an earnest evangelical and a practical philanthropist, "ever ready with his sympathy, his time, and his money to promote any desirable object." Born at Margate, a scholar of Lincoln College, Oxford, who subsequently took a First in Greats, Dr. Miller passed from a curacy at Park Chapel, Chelsea, to the rectory of St. Martin's, Birmingham, in 1846. The selection, under a remarkable trust, was a tribute to Dr. Miller's piety. Twenty years of his ministry were spent at this parish, the organisation of which came to be widely admired. He helped to found a Working Men's Association, and was active in convocation, but he became most widely known as the successful pioneer of Hospital Sunday.

### The First Hospital Sunday.

In 1859 the Birmingham General Hospital was very short of money, and it was on October 13 of that year that Canon Miller unfolded in the Press the plan for a simultaneous collection on a given Sunday in every place of worship for the hospital. In this public letter Canon Miller wrote: "I know that the ministers of religion will find a difficulty in agreeing upon the day. But much of the efficiency of the suggestion lies in this. Let the collection come in driblets, and much of the spirit of the movement is lost." Within a month of this suggestion the first Hospital Sunday was held in Birmingham, and more than £5,000 was collected for the General Hospital. The movement spread rapidly to other towns, and eventually to the Colonies, for its first supporters, the clergy themselves, saw in Hospital Sunday not only co-operation in a work of mercy, but the value of a common meeting-place for all denominations. In these days, when "the exchange of pulpits" is often recommended, the earliest expression of such inter-relation is worth remembering. In 1866 the Prime Minister, Lord Russell, presented Dr. Miller to the vicarage of Greenwich, where he remained till his death in 1880. In an interesting letter dated July 19, 1875, preserved among the late Sir Henry Burdett's papers, the Rev. John Henn, then secretary to the Manchester and Salford Saturday and Sunday Funds, referred to Canon Miller, "who began what he called 'periodical collections for local charities' in Birmingham in 1858. It was on the model of this I started (1870) our Hospital Sunday here, and all the rest of the Hospital Sundays are copies of ours."

This claim for Manchester is historically interesting, and is also a tribute to Canon Miller for having started an invaluable principle, which holds its definite place to-day.

**The Story of Hospital Sunday**—(*continued*).

Enter Sir Sydney Waterlow.

Though Hospital Sunday collections were made in Bristol for the first time in 1859, the movement took thirteen years to reach London, and how it fared in the City is recorded in *Burdett's Hospitals and Charities* for 1899. In 1873 the Lord Mayor of London was Sir Sydney Waterlow. Anxious to start Hospital Sunday in London, Sir Sydney wrote to the Lord Mayor of Birmingham for particulars of the organisation and method adopted. "Sir Sydney's letter was handed by the Lord Mayor of Birmingham to the author of *Burdett's Hospitals and Charities*, and he prepared a statement and supplied all forms, with the aid of which Sir Sydney Waterlow instituted the Hospital Sunday Fund in London."

It had been Canon Miller's success in Birmingham which inspired Mr. Ramsay, then secretary of St. Mark's Hospital, to hope that the movement would be transplanted to London. His hope began to be realised when, at the London Tavern, Bishopsgate Street, twenty-four of the chief London hospitals were represented in an assembly held on November 21, 1872. The Bishop of London promised to invite the help of the Rural Deans, and the Lord Mayor, Sir Sydney Waterlow, was asked to preside at a public meeting. He was enthusiastic, and became President and Treasurer, a post which his successors at the Mansion House have continued to hold. The editor of the *Lancet*, Dr. James Wakley, also gave valuable help, and the movement gradually triumphed over the denominational difficulties which first threatened it. The first Council was elected in January 1873, and the June 15 was the date of the first Hospital Sunday in London. The first Council of the Fund included Miss Florence Nightingale, Mrs. Garrett Anderson, Lady Burdett Coutts and Miss Gladstone.

SIR SYDNEY H. WATERLOW, First President of the Metropolitan Hospital Sunday Fund and Vice-President, 1874-1905.

Dr. Wakley's Move in 1886.

The result was that 1,072 congregations contributed, and a sum of £27,700 was raised. The total on the whole, declined till 1878, when the receipts began to rise till, in 1884, £39,330 was raised by 1,522 congregations. In 1885 there was another fall, which led Dr. James Wakley, then editor of the *Lancet*, to publish in the following year a special Supplement, edited by the late Sir Henry Burdett, which raised the receipts in 1886 to £40,399, although only 1,595 congregations contributed. The outcome of this Supplement was The Hospital newspaper, which appeared independently in October 2, 1886, "to stimulate the interest of the public in the voluntary system of hospital support and to deal with all questions involved in the maintenance of an adequate hospital system." Dr. Wakley died in 1887, and The Hospital to this day has continued the publication of a special supplement devoted to Hospital Sunday which the *Lancet* had begun.

The Triumph of 1895.

We now come to a period in which the total receipts of the Hospital Sunday Fund exceeded £40,000 until 1892, thanks, however, to the generous help of certain enthusiasts. Among these were the late Duke of Cleveland and Lord Somerleyton, who, in 1891, when Sir Savile Crossley, Bart., gave £1,000, which became an annual subscription with him till 1896. Bequests also came to be made, but these and the assiduity with which the then Secretary of the Fund, Mr. Henry N. Custance, succeeded in raising the number of contributing congregations to some 1,800, failed to keep the annual income at its previous height. This declension was met by a special effort in 1895, when The Hospital led a newspaper campaign which, for that year, raised the total to £60,000, a figure which roughly represents the number of copies of our Supplement that were then circulated. It is recorded that £18,000 was sent in contributions to the Lord Mayor in consequence of The Hospital's appeal. One-third of the total came from legacies and special donations, including £3,400 from members of the Stock Exchange. Till 1899 the previous average of £40,000 was not much

**The Story of Hospital Sunday**—(*continued*).

increased, except that in 1899 itself Mr. George Herring made his splendid gift of £10,000, which raised the total to over £50,000 once more.

The Link with the Mansion House.

In him and in others the Fund discovered firm friends, some of whom are commemorated in the illustrations published in this number. Sir Sydney Waterlow, for instance, not only instituted the Fund

MR. GEORGE HERRING.

in London, but was its Vice-President from the start, and, till 1899 at any rate, never missed being present to move the adoption of the Distribution Committee's report at the annual meeting. The progress which the Fund made in these twenty-five years (1874-99) was evidence of his leadership; and sincere thanks must be given to the successive Lord Mayors who have been Presidents of the Fund during their year of office, and have often added to their official duties much personal work in its behalf. The Fund's link with the Mansion House cannot be too much appreciated.

The financial story comes to this: that Hospital Sunday in London raises now in rough figures £86,000 of which £46,000 or one-half is derived from legacies and investments. As we show in our article on p. 313 this contents no one, but the history of the Fund proves that the means which has always worked wonders before must be invoked again. The remedy is for a new Canon Miller, a new Dr. Wakley—for a man, in short, with sufficient belief in Hospital Sunday to infect the clergy with his own enthusiasm.

"We cannot help thinking," wrote the Editor of *Hospitals and Charities* in 1914, "that the original object of the Fund, *viz.*, to benefit the hospitals, has, to a certain extent, been lost sight of, and that many of the clergy and ministers look upon the fund merely as a centre for the distribution of hospital letters and the supply of surgical appliances. Hence the original motive and energy have been sadly lacking in recent years." So just do we think this criticism that the "original motive" is re-stated as boldly as possible in our first article this week in the Hospital Sunday Fund Section. The answer to those who regard charity as a business, to be run on business lines, is that, when it is so treated, support weakens, and can be regained only when a disinterested motive once more finds a sincere spokesman.

The business side of the Fund is its method of distribution. Because it was difficult to determine the due claim of each hospital merely by its own presentment of accounts, the Fund mended matters by insisting upon the adoption of the Uniform System of Accounts, which came into force in 1892, and has not only made correct comparisons possible, but maintained some check upon inefficiency.

Another general advantage encouraged by the Fund has been the installation of a Lady Almoner at each up-to-date hospital. This officer, it is important to remember, was installed at first merely

DR. JAMES WAKLEY.

to prevent the abuse of the out-patient departments by persons who could afford to pay and therefore were not entitled to free treatment. But the voluntary system quickly humanised this department, so that the Inquiry Officer soon developed into a Lady Almoner, whose main duties were more inviting. Thus every subscriber on Hospital Sunday should remember that his gift does not end with the money-value of his contribution, but helps in a hundred humane ways the sick and needy, and strengthens the whole voluntary system through the Hospital Sunday Fund.

**The Story of Hospital Sunday—(*continued*).**

Facts like these the clergy themselves should master, that they may instil into their congregations that lesson of personal service on the part of the healthy for the sick which is the mainspring of the whole movement? In so far as this motive is ignored has the Fund stood still or declined. In so far as this motive has been remembered has the Fund advanced toward the £100,000 a year, which was its original aim in London.

The ups and downs of finance have not been the only incidents in the Fund's story. There have been lively controversies and animated debates, "packed meetings" and all the manifestations which accompany the pains of growth. It was only with difficulty that the Fund achieved a constitution. In the early days the results of the annual collections and the reports of the council were made public at a meeting in the Egyptian Hall of the Mansion House, which it was open to anyone to attend. The consequence was that when the Distribution Committee made its award "on the basis of merit" as well as on the turnover represented by expenditure, any hospital which looked to the Fund to supply deficiencies (which, perhaps, better management could have removed), had only to muster sufficient supporters to "pack" the Mansion House meetings. When a special representative committee was appointed "to prepare a system of distribution providing for the points of dispute" there was much opposition from the representatives of certain special hospitals, led by the late Sir Morrell Mackenzie. Their claims were investigated by a committee appointed on the motion of Cardinal Manning.

Its suggestions were considered on March 8, 1875, in the Egyptian Hall at the Mansion House. The committee recommended that so long as hospitals, general or special, were well-conducted, there should be no difference between them in the basis of award. Dr. Morrell Mackenzie moved an amendment "to render ineligible to serve on the Distribution Committee any person holding office in a hospital or dispensary, and to place dispensaries on the same footing as hospitals in regard to the general basis of award, so long as they were managed by a committee." His amendment, in the words of *Hospitals and Charities* (1913) "was carried by an overwhelming majority, owing to the fact that the Egyptian Hall was crowded with the out-patients of certain special hospitals who came there with the sole object of supporting the mover of the amendment." The result was the holding of a meeting of the clergy and ministers and lay representatives of the congregations at the Mansion House on May 7, 1875, when the constitution of the Fund, substantially as it now exists, was adopted. On June 20, 1913 the revised laws of the constitution were adopted by a meeting of the constituents at the Mansion House, which are set forth in the 1914 issue of *Hospitals and Charities*.

Thus in outline is the story of the movement which Canon Miller founded sixty-one years ago. It started in the provinces, and after London was enrolled it has continued to spread. Its ups and downs have been of the same character everywhere. But behind them remains still the original motive and intention, and only by reference to them can the successes and relapses of the movement be understood. The story is worth telling for its own sake, and because the popular love of new truths is so often purchased by a forgetfulness of old ones. But our first duty is to be true to the great men and movements of the past.

## Figures and Tables

**Figure f1:**
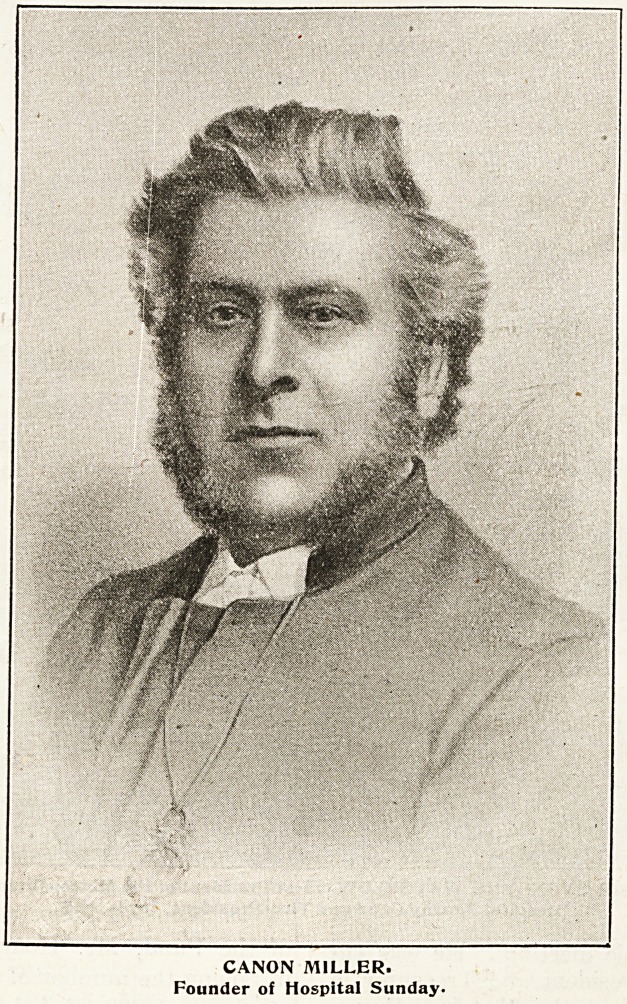


**Figure f2:**
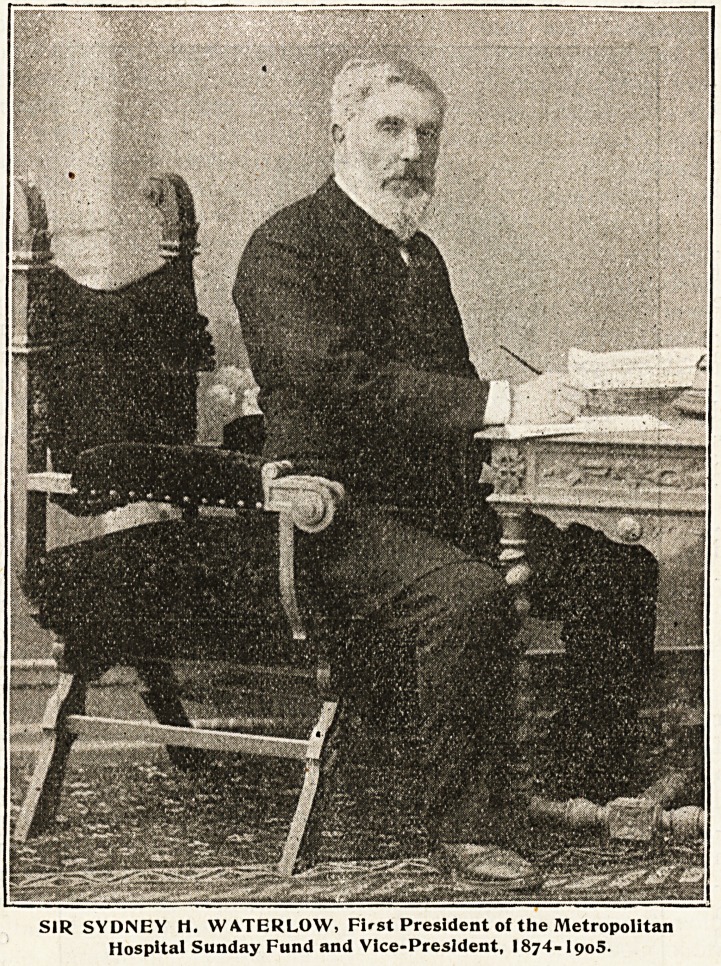


**Figure f3:**
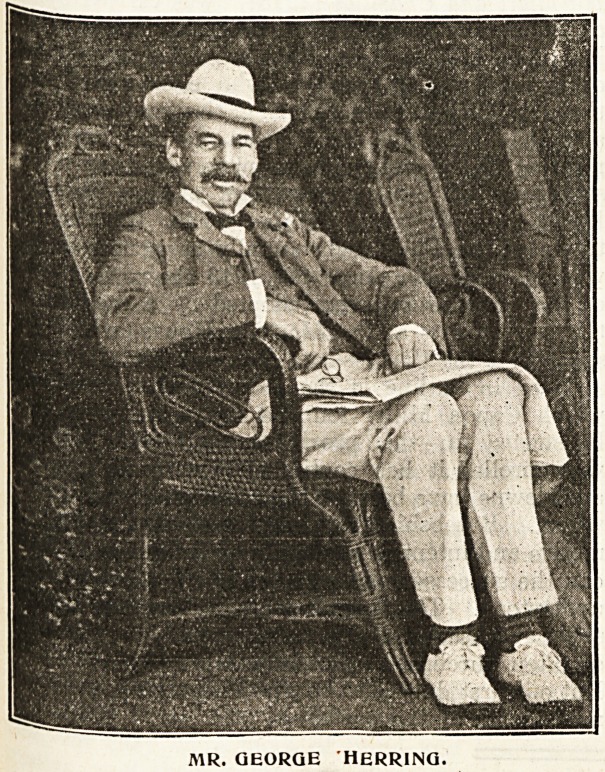


**Figure f4:**